# Using surrogate vaccines to assess feasibility and acceptability of future HIV vaccine trials in men: a randomised trial in inner-city Johannesburg, South Africa

**DOI:** 10.1186/s12889-017-4355-z

**Published:** 2017-07-04

**Authors:** Lucy Chimoyi, Mphatso Kamndaya, Emilie Venables, Nina von Knorring, Jonathan Stadler, Catherine MacPhail, Matthew F. Chersich, Helen Rees, Sinead Delany-Moretlwe

**Affiliations:** 1 Wits RHI, Faculty of Health Sciences, University of the Witwatersrand, Johannesburg, South Africa; 2 Division of Social and Behavioural Sciences, School of Public Health and Family Medicine, University of Cape Town, Cape Town, South Africa; 3 Clinical Microbiology and Infectious Diseases, Faculty of Health Sciences, University of the Witwatersrand, Johannesburg, South Africa; 4 School of Health and Society, University of Wollongong, Wollongong, NSW Australia

**Keywords:** HIV vaccine, Trial, Men, Feasibility, Acceptability, Sub-Saharan Africa, Surrogate vaccine

## Abstract

**Background:**

Developing an effective HIV vaccine is the overriding priority for HIV prevention research. Enrolling and maintaining cohorts of men into HIV vaccine efficacy trials is a necessary prerequisite for the development and licensure of a safe and efficacious vaccine.

**Methods:**

One hundred-fifty consenting HIV-negative men were enrolled into a pilot 1:1 randomised controlled trial of immediate vaccination with a three-dose hepatitis B vaccine compared to deferred vaccination (at 12 months) to investigate feasibility and acceptability of a future HIV vaccine trial in this population. Adverse events, changes in risk behaviour, acceptability of trial procedures and motivations for participation in future trials were assessed.

**Results:**

Men were a median 25 years old (inter-quartile range = 23–29), 53% were employed, 90% secondary school educated and 67% uncircumcised. Of the 900 scheduled study visits, 90% were completed in the immediate vaccination arm (405/450) and 88% (396/450) in the delayed arm (*P* = 0.338). Acceptability of trial procedures and services was very high overall. However, only 65% of the deferred group strongly liked being randomised compared to 90% in the immediate group (*P* = 0.001). Informed consent processes were viewed favourably by 92% of the delayed and 82% of the immediate group (*P* = 0.080). Good quality health services, especially if provided by a male nurse, were rated highly. Even though almost all participants had some concern about the safety of a future HIV vaccine (98%), the majority were willing to participate in a future trial. Future trial participation would be motivated mainly by the potential for accessing an effective vaccine (81%) and altruism (75%), rather than by reimbursement incentives (2%).

**Conclusions:**

Recruitment and retention of men into vaccine trials is feasible and acceptable in our setting. Findings from this surrogate vaccine trial show a high willingness to participate in future HIV vaccine trials. While access to potentially effective vaccines is important, quality health services are an equally compelling incentive for enrolment.

**Electronic supplementary material:**

The online version of this article (doi:10.1186/s12889-017-4355-z) contains supplementary material, which is available to authorized users.

## Background

In 2015, there were 37 million people estimated to be living with HIV globally; 26 million in sub-Saharan Africa (SSA), of which 10 million were men [1]. Despite the scaling up of access to HIV testing, condoms, voluntary medical male circumcision and antiretroviral treatment (ART), 2 million new infections occurred in 2015, 68% in SSA [1–4]. An effective HIV vaccine represents the best long-term hope for controlling the pandemic [3, 5, 6], but several features of the virus, including its rapid mutation and multiple clades, pose considerable challenges to the development of an efficacious vaccine [7, 8]. Despite this, the RV144 Thai phase III HIV vaccine trial using two booster injections in addition to a recombinant canarypox vector vaccine showed a modest reduction in HIV infections [9]. Further trials of this approach are planned for Southern Africa, with the aim of extending the RV144 results and ultimately of vaccine licensure [7, 8].

Conducting large phase III HIV prevention trials is challenging, however, and relatively little is known about men’s participation in HIV prevention research [10]. Most HIV prevention interventions investigated so far have been targeted at women, who have a higher risk for infection and a compelling need for female-controlled methods of HIV prevention [11–13]. In this context, research is usually focussed only indirectly on men in their role as sexual partners of women, rather than as actual participants in a trial [14]. Evaluation and licensure of an HIV vaccine will, however, require the enrolment of large cohorts of men at risk for HIV infection, given, among other reasons, that immune responses to vaccines vary by gender [15].

Current evidence for HIV vaccine acceptability and trial feasibility comes largely from studies focussing on women [16, 17], people who inject drugs [18], men who have sex with men [19], discordant couples [16] and male army conscripts [20–22]. There are clearly evidence gaps around the feasibility of HIV vaccine trials among heterosexual men in SSA. Vaccine preparedness studies that have explored willingness to participate (WTP) in HIV vaccine trials have been conducted without using an actual vaccine or a clinical trial design [23–25]. Giving an actual vaccine – even if not an HIV vaccine – would more closely approximate an HIV vaccine trial, enabling investigation of trial procedures, such as randomisation, the provision of repeated vaccine doses and the monitoring of adverse events. Using a trial design also allows for assessment of the perspectives of men towards being randomised and how that process is explained during informed consent. Furthermore, understanding whether trial participation is driven by a desire for access to a vaccine perceived as protective, the clinical attention received as a trial participant, or the trial as a whole is important. These questions are best answered in people who have actually participated in a trial, rather than by eliciting hypothetical views.

Recent experiences of suboptimal adherence to potentially efficacious interventions in HIV prevention trials have provided a timely reminder about the importance of including end-user perspectives in the design and delivery of prevention products [26, 27]. We conducted a pilot randomised controlled trial using a hepatitis B vaccination as a surrogate for an HIV vaccine in high-risk HIV-negative men in Johannesburg, South Africa. We evaluated the recruitment rate, retention, adverse events, behaviour changes and willingness to participate in future HIV vaccine trials, and specifically explored whether participation in a trial would be motivated by access to the vaccine, or to the clinical care and the trial as a whole.

## Methods

### Study population

From August to September 2011, participants were recruited from primary health care clinics in Johannesburg Region F, male-oriented venues in the community and referrals from enrolled participants. The following clinics were involved: Esselen, Jeppe Joubert Park, Malvern, Marshalls Town, Mayfair, Rosettenville and Yeoville clinics, and the Hillbrow Community Health Centre. Men were considered eligible for enrolment if older than 18 years, sexually active (had sex in past 3 months), HIV negative, negative for hepatitis B core antigen and hepatitis B surface antigen (chemiluminescent microparticle immunoassay, Abbott Architect i2000), and if they had no clinical evidence of chronic hepatitis B infection. Men with a history of bleeding disorders, hypersensitivity to the hepatitis B vaccine or its ingredients, and those with evidence of acute infection or a fever >37.8 °C were excluded. Those excluded for medical conditions were referred to local health care facilities for further management and care.

### Randomisation and study procedures

Eligible participants who demonstrated adequate understanding of trial procedures through a comprehension checklist were enrolled after providing written informed consent for study participation and for long-term storage of biological specimens. Informed consent procedures included a thorough explanation of randomisation concepts, study visit schedules and encouragement of participants to discuss any concerns about the study. A pre-vaccination assessment, including physical and genital examination, was performed for evidence of sexually transmitted infections (STIs).

Sequentially numbered envelopes containing study allocation were pre-prepared by an independent statistician based on simple randomisation using a random number list. Envelopes were assigned consecutively and opened in the presence of the participant. Participants were randomised 1:1 to receive immediate vaccination (IV) or deferred vaccination (DV). The hepatitis B vaccine ENGERIX-B or equivalent generic from the GSK Biologicals was administered in three doses during the study in the IV group (at enrolment, and at months 1 and 6). Participants in the DV group were offered vaccination at the month 12 visit, again given in three doses. In accordance with manufacturer’s instructions, 1-mL adult dose was provided, that contains 20 mcg of hepatitis B surface antigen adsorbed on 0.5 mg aluminium as aluminium hydroxide. The adult formulation contained sodium chloride (9 mg/mL) and phosphate buffers (disodium phosphate dihydrate, 0.98 mg/mL; sodium dihydrogen phosphate dihydrate, 0.71 mg/mL). Vaccination was deferred to the next visit in those that had evidence of an acute febrile illness.

The hepatitis B vaccine was selected as it shares some features with a potential HIV vaccine: the infective agent in both cases is a virus that causes chronic infection and shares transmission modes, and the hepatitis B’ vaccine’s multi-dose regimen is likely analogous to that of a future HIV vaccine. Deferring vaccination, rather than administering a placebo vaccine, for example, enabled us to compare the hypothetical perspectives and adverse events between the groups. Relative rates of adverse events in the two groups may provide useful information on the expected rates of vaccine site soreness or swelling in future HIV vaccine trials, for example. Lastly, providing a vaccine after study completion may actually occur in HIV vaccine trials, where participants in a placebo arm may be invited to receive the HIV vaccine if it were shown to be effective.

Following randomisation, participants were seen at monthly intervals for the first 3 months of follow-up, and then quarterly until month 12. A structured interviewer-administered questionnaire was administered at baseline and every 3 months thereafter. At screening for study enrolment and each quarterly visit, participants were also counselled and tested for HIV using Determine HIV-1/2 rapid tests (Alere Determine^TM^), followed by Unigold Recombigen^TM^. Discordant results were confirmed using Vironostika HIV-1 ELISA testing. Blood samples at baseline were also tested for syphilis (rapid plasma reagin and *Treponema pallidum* particle agglutination tests) and HSV-2 (HerpeSelect HSV-2 ELISA IgG; Focus Diagnostics). Urine samples were tested for *N. gonorrhoeae, C. trachomatis* and *T. vaginalis* using a multiplex PCR (Seeplex® STI Master Panel 1, 2, 3).

During interviews, data were collected on participants’ socio-demographics, sexual behaviours and medical history. Post-vaccination assessments for adverse events were performed 30 min after vaccination and 1 month thereafter. To allow for comparison of the rates of adverse events between the two study arms, we collected data on adverse events in all participants at enrolment, and at months 1, 2, 6, 7. Adverse events at enrolment were collected 30 min after vaccination in the IV group and after randomisation in the DV group for comparative purposes. At all visits, participants received risk-reduction counselling, free condoms, and STI treatment if required (syndromic for symptomatic STIs or treatment for asymptomatic curable infections diagnosed with laboratory testing). Those testing positive for HIV during the trial were retained in the study, and referred to local HIV treatment facilities for further care.

At month 12, to assess acceptability of participation in the current trial and in future HIV vaccine trials, we collected data on participants’ perspectives on several aspects of the study procedures (10 items) and clinical services they had received (10 items). In these measures, participants were asked to rate how they felt about the procedure or service on a scale of 0–5 (with 0 being those least liked and 5 being most liked).

### Outcome measures and analysis

The primary outcomes were the proportion of men screened who fulfilled all eligibility criteria and accepted enrolment; the number of expected visits actually attended; and the proportion successfully retained in the trial at 12 months. We also calculated the ratio of screened-to-enrolled men and used Generalised Estimating Equations to identify factors associated with retention. Model selection was carried out beginning with the set of significant univariate predictors (*P* < 0.1) and model fit assessed using goodness of fit tests. The variables study group (DV and IV) and age were forced into the model. In a separate univariate analysis, we also explored whether any of the 20 acceptability items were associated with retention. In this exploratory analysis we defined retention as having attended all four visits (acceptability was asked at month 12, so this analysis only included those who had attended that visit).

Vaccine dose completion and frequency of AEs were considered additional measures of trial feasibility. In the IV group, AEs occurring 30 min after vaccine administration or up to 1 month later were considered post-vaccination events. For analysis of acceptability, we compared the proportion in each study arm who rated an item as a 4 or 5. WTP was assessed at month 12 by the proportion who said they would join a future HIV vaccine trial. Participants were also asked if they would want to receive an HIV vaccine, should it be shown to be effective, approved and registered. Finally, we explored the phenomenon of risk compensation by comparing sexual behaviours in each group (condom use, alcohol intoxication during sex, number of partners and having an HIV-positive partner) at quarterly visits. HIV incidence and the point prevalence of STI episodes were estimated in the overall cohort. Differences between categorical variables were examined using the chi-square test or Fisher’s exact test when the expected values in any of the cells was below five [28]. Wilcoxon Rank Sum tests were used for comparing continuous data. All analyses were performed using Stata^TM^ Version 12.

## Results

### Participant characteristics

**Fig. 1:**
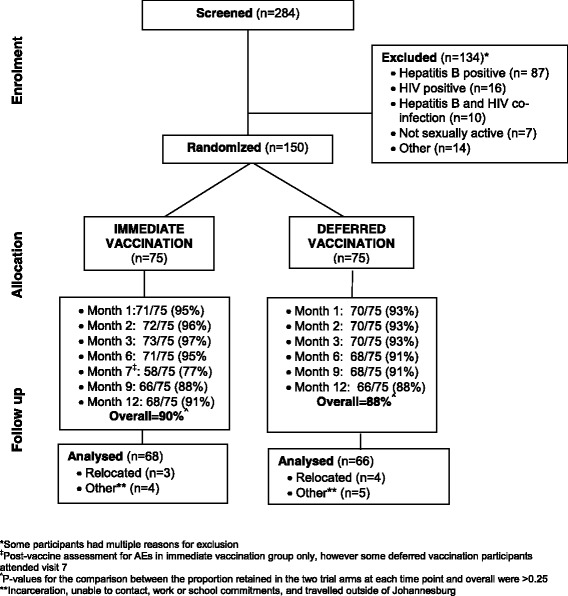
CONSORT Diagram – Flow of participants through trial period

In total, 284 men were screened and 150 then randomised to receive immediate (*n* = 75) or deferred (*n* = 75) vaccination (screen-to-enrol ratio = 1.89). The main reasons for non-enrolment were positive hepatitis B serology or HIV infection (Fig. 1). Compared to those enrolled, non-enrolled men were older, more likely to be married, and reported lower rates of condom use and more lifetime partners (Table 1). A quarter of non-enrolled men perceived themselves to be at high-risk for HIV, consistent with the prevalence of HIV (12%), reactive syphilis serology (12%) and HSV-2 (41%) in these men. *C. trachomatis* infection rates were high in both study arms and the non-enrolled men (6.7–9.0%).

**Table 1:** Baseline characteristics of men, comparison by enrolment status and study arm

Variable	Enrolled			Not enrolled (C) (*n* = 134)	*P* (A + B vs C)
	Immediate (A) (*n* = 75)	Deferred (B) (*n* = 75)	*P* (A vs B)		
Median age (IQR)	25 (23–29)	26 (23–29)	0.390	30 (26–36)	<0.001
Born in SA	50 (66.7)	49 (65.3)	0.864	93 (69.4)	0.541
Single	64 (85.3)	64 (85.3)	1.000	102 (76.1)	0.002
Any employment^a^	40 (53.3)	40 (53.3)	1.000	78 (58.2)	0.409
Completed secondary education	72 (96.0)	63 (84.0)	0.018	115 (85.8)	0.482
Johannesburg resident >1 year	69 (92.0)	67 (89.3)	0.575	120 (89.6)	0.530
Median lifetime partners (IQR)	7 (5–13)	9 (5–20)	0.238	10 (6–20)	0.007
Median age coital debut (IQR)	16 (15–18)	16 (15–19)	0.861	17 (15–19)	0.132
Used condom at each sex act^b^	47 (62.7)	58 (77.3)	0.063	45 (52.3)	0.052
High-risk female partners^c^	33 (44.0)	31 (41.3)	0.741	62 (51.7)	0.767
Vaccination history^d^	48 (64.0)	55 (73.3)	0.309	106 (79.1)	0.126
Perceived HIV risk	10 (13.3)	6 (8.0)	0.551	32 (23.9)	0.010
Previous sex with male partner	2 (2.7)	1 (1.3)	0.500	4 (3.0)	0.710
Circumcised	22 (29.3)	27 (36.0)	0.384	48 (35.8)	0.545
Syphilis^e^	2 (2.7)	2 (2.7)	0.690	16 (11.9)	<0.001
*N. gonorrhoea* ^e^	2 (2.7)	1 (1.3)	0.500	2 (1.5)	0.553
*T. vaginalis* ^e^	1 (1.3)	0 (0.0)	-	6 (4.5)	0.055
*C. trachomatis*	10 (6.7)	12 (8.0)	0.907	12 (9.0)	0.139
HSV-2	17 (22.7)	19 (25.3)	0.512	54 (41.2)	<0.001
HIV-1	0 (0.0)	0 (0.0)	-	16 (12.0)	-
Hepatitis B	0 (0.0)	0 (0.0)	-	87 (65)	-
HIV and hepatitis co-infection	0 (0.0)	0 (0.0)	-	10 (7)	-

^a^Part-time, self- or full-employment; ^b^In past 3 months, with most recent partner; ^c^Sex partner has other partners and never tested for HIV to man’s knowledge; ^d^Any vaccination, including experimental or immunisations. ^e^Fisher’s exact test

Baseline characteristics of participants in the two trial arms were similar (Table 1). Enrolled participants were a median 25 years (inter-quartile range [IQR] = 23–29). The majority were single (85%), secondary school educated (90%), employed (53%) and resident in Johannesburg for more than a year (91%). Median age at sexual debut was 16 years (IQR = 15–19) and men had a median 8 lifetime partners (IQR = 5–15). High baseline condom use (70%) and low levels of circumcision (33%) characterised this population. HSV-2 prevalence was 24% in the enrolled participants.

### Feasibility and study retention

Overall, 801/900 (89%) of the expected visits were completed (405/450 [90%] IV vs. 396/450 [88%] DV, *P* = 0.338; Fig. 1). Median follow-up time was 11.8 months in both groups. Seven in the IV group and 9 in the DV group did not attend the month 12 visit, despite repeated attempts to trace them (2 in IV and 3 in DV could not be contacted). Other reasons for participant loss included travel away from the area (IV = 1 vs. DV = 2), relocation (IV = 1 vs. DV = 2), inability to attend due to work or school commitments (IV = 2 vs. DV = 1) and incarceration (IV = 1 vs. DV = 1). In multivariate analysis (Additional file 1: Table S1), no difference was detected between retention in the two study arms (adjusted OR comparing DV to IV = 1.36; 95% CI = 0.56–3.35). The odds of retention in men who had an adverse event were 3.76 that of other men (95% CI adjusted OR = 0.82–17.14). Age was not associated with retention.

### Vaccine completion

In the IV group, no participants refused vaccination post-enrolment, but 5% missed dose 2 (4/75) and 8% dose 3 (6/75). Two had vaccinations deferred until the next visit, one with a contra-indication (flu) and one who missed the scheduled visit. Dose completion in the IV group was more frequent in men reporting consistent than inconsistent condom use in the 3 months prior to baseline (94% vs. 75%, *P* = 0.022), and in those who had lived in Johannesburg for over a year, compared to briefer periods (93% vs. 67%, *P* = 0.006).

At study end, 61% (40/66) of participants in the DV group returned for hepatitis B vaccination. Reasons given for non-acceptance included unwillingness to be vaccinated (6) and current receipt of TB treatment (1). No differences were noted in the sociodemographic characteristics or acceptability measures between those in the DV group who were or were not vaccinated.

Overall, 61 AEs were reported. All AEs were non severe (Grade 1) and no allergic reactions related to vaccination were reported. More AE were reported by the IV than DV study arm (40 versus 21, *p* ≤ 0.001; Table 2). Much of this difference is accounted for by injection site soreness or swelling in the IV group (12). In the IV group, 17% reported an AE after the month 1 vaccine and 13% after the month 6 vaccine.

**Table 2:** Frequency of adverse events at enrolment, and months 1, 2, 6 and 7, by study arm (total visits = 600)

Type of event	Enrolment (*n* = 150) n events (%)		Month 1 (pre-vaccination), 2 (post-vaccination) (*n* = 141) n events (%)				Month 6 (pre- vaccination), 7 (post- vaccination) (*n* = 139) n events (%)				Total N events (%)
	IV (*n* = 75)	DV (*n* = 75)	IV (*n* = 71)		DV (*n* = 70)		IV (*n* = 71)		DV (*n* = 68)		
			Pre	Post	‘Pre’	‘Post’	Pre	Post	‘Pre’	‘Post’	
Chills	0 (0.0)	0 (0.0)	0 (0.0)	0 (0.0)	2 (2.7)	3 (4.1)	2 (2.7)	1 (1.3)	1 (1.4)	1 (1.4)	10 (0.7)
Headache	0 (0.0)	2 (2.7)	1 (1.3)	3 (4.0)	3 (4.1)	0 (0.0)	3 (4.0)	0 (0.0)	0 (0.0)	1 (1.4)	13 (1.0)
Nausea	2 (2.7)	1 (1.4)	0 (0.0)	1 (1.3)	0 (0.0)	0 (0.0)	1 (1.3)	0 (0.0)	0 (0.0)	0 (0.0)	5 (0.4)
Fatigue	0 (0.0)	0 (0.0)	1 (1.3)	1 (1.3)	1 (1.4)	0 (0.0)	1 (1.3)	0 (0.0)	0 (0.0)	0 (0.0)	5 (0.4)
Limited ADL^c^	0 (0.0)	0 (0.0)	1 (1.3)	0 (0.0)	0 (0.0)	0 (0.0)	0 (0.0)	1 (1.3)	0 (0.0)	0 (0.0)	2 (0.2)
Dizziness	0 (0.0)	0 (0.0)	0 (0.0)	1 (1.3)	2 (2.7)	0 (0.0)	0 (0.0)	0 (0.0)	0 (0.0)	0 (0.0)	3 (0.2)
Injection site soreness	0 (0.0)	0 (0.0)	1 (1.3)	3 (4.0)	0 (0.0)	0 (0.0)	0 (0.0)	6 (8.0)	0 (0.0)	0 (0.0)	10 (0.7)
Injection site swelling	0 (0.0)	0 (0.0)	1 (1.3)	1 (1.3)	0 (0.0)	0 (0.0)	0 (0.0)	0 (0.0)	0 (0.0)	0 (0.0)	2 (0.2)
Allergic reaction	0 (0.0)	0 (0.0)	0 (0.0)	0 (0.0)	0 (0.0)	0 (0.0)	0 (0.0)	0 (0.0)	0 (0.0)	0 (0.0)	0 (0.0)
Other^b^	5 (6.7)	4 (5.4)	0 (0.0)	2 (2.7)	0 (0.0)	0 (0.0)	0 (0.0)	1 (1.3)	0 (0.0)	0 (0.0)	11 (0.8)
Total	7 (9.3)	7 (9.3)	5 (7.0)	12 (16.9)	8 (11.4)	3 (4.3)	7 (9.9)	9 (12.7)	1 (1.5)	2 (2.9)	61 (10.2)^a^

^a^AEs collected at total of 600 visits; Post-vaccination events in IV arm include events 30 mins or 1 month after vaccination; DV arm ‘pre’ AEs are those recorded at time of visit, and ‘post’ are AEs at visit 1 month later.^b^Other reported AEs include: cough, nights sweats and tonsillitis; ^c^Activities of daily living. Fisher’s exact test done to compare groups, all *P* values were >0

Five HIV infections (1 in IV and 4 in DV group) were observed over the 1598.3 person-months of follow up (HIV incidence 0.3/100 person months; 95% CI: 0.2–0.9). No incident cases of hepatitis occurred during the trial. All five infections occurred in the first 6 months of follow-up.

### Changes in risk behaviour

In both study groups, men reported a higher coital frequency and lower condom use at baseline than at all subsequent visits (Table 3). Compared to the IV group, a higher proportion of DV participants reported sex with a condom at each study visit, including at enrolment. The DV group also had more alcohol-intoxicated sexual acts at each visit than the IV group. However, neither condom use nor intoxication differences were significant. Few men reported having sex with a partner known to be HIV positive at enrolment, and levels were even lower at subsequent visits (*P* = 0.061). At study end, participants rated the validity of their responses to self-reported questions, scoring themselves relatively high with condom use (IV 60 [88%] vs. DV 56 [85%]), number of partners (IV 65 [96%] vs. DV 58 [88%]) and sexual behaviour in general (IV 67 [89%] vs. DV 65 [98%]).

**Table 3:** Changes over time in risk behaviour, symptoms of sexually transmitted infection and HIV incidence, by group

Behaviour	Group	Baseline (*n* = 150)	Month 3 (*n* = 143)	Month 6 (*n* = 139)	Month 9 (*n* = 134)	Month 12 (*n* = 135)	*P* ^#^
Median sexual partners in past 3 months	IV	1 (1–2)	1 (1–2)	1 (1–2)	1 (1–2)	1 (1–2)	0.993
	DV	1 (1–2)	1 (1–2)	1 (1–2)	1 (1–2)	1 (1–2)	
	P*	0.769	0.644	0.685	0.959	0.558	
Median sexual acts in past 3 months	IV	9 (3–21)	6 (2–12)	5.5 (2–12)	5 (2–10)	6 (3–15)	-
	DV	9 (3–20)	6 (3–14)	6 (3–11)	6 (3–11)	6 (3–12)	
	P*	0.795	0.339	0.392	0.489	0.972	
Condom use in past 3 months	IV	55 (73.3%)	56 (81.1%)	55 (80.9%)	50 (79.4%)	52 (81.3%)	0.461
	DV	62 (82.7%)	60 (87.0%)	57 (85.1%)	58 (86.6%)	55 (84.6%)	
	P*	0.168	0.352	0.517	0.274	0.611	
HIV-positive sex-partner^a, c^	IV	2 (2.7%)	0 (0.0%)	0 (0.0%)	0 (0%)	0 (0.0%)	0.061
	DV	3 (4.0%)	1 (1.3%)	2 (2.7%)	1 (1.3%)	2 (2.7%)	
	P*	0.500	0.500	0.248	0.500	0.248	
Sexual acts while intoxicated in past 3 months	IV	12 (16.0%)	16 (21.3%)	13 (17.3%)	17 (22.7%)	18 (24.0%)	0.233
	DV	18 (24.0%)	19 (25.3%)	18 (24.0%)	20 (26.7%)	19 (25.3%)	
	P*	0.221	0.562	0.313	0.570	0.850	
Incident HIV infection^b, c^	IV	-	0 (0.0%)	0 (0.0%)	0 (0.0%)	0 (0.0%)	0.299
	DV	-	2 (2.7%)	1 (1.4%)	0 (0.0%)	0 (0.0%)	
	P*	-	0.245	0.497	-	-	

^a^Where participant knows partner’s HIV status; *P value compares groups at every month; ^#^ *P* value across visits overall; ^b^2 incident HIV infections at Month 2 in IV group; ^c^Fisher’s exact test

### Acceptability

Of the study procedures assessed, more than 90% of participants rated questionnaire completion, repeated HIV testing and receipt of reimbursements as a 4 or 5 out of 5 (Table 4). Only 65% of the deferred group, however, gave high ratings for being randomised, compared to 90% of the immediate group (*P* = 0.001). By contrast, 92% of the DV group held favourable views on the informed consent processes, compared to 82% of the IV group (*P* = 0.080). The collection of blood and genital specimens were viewed relatively unfavourably by both groups.

**Table 4:** The proportion of participants at month 12 who felt they liked the study procedures and clinical services, by study arm (*n* = 134)

Item	Immediate vaccination (*n* = 68) n liked or very liked (%)	Deferred vaccination (*n* = 66) n liked or very liked (%)	*P*
Study procedures			
Informed consent	56 (82.4)	61 (92.4)	0.080
Randomisation	61 (89.7)	43 (65.2)	0.001
Completing questionnaires	62 (92.5)	61 (92.4)	0.980
Physical examination	60 (88.2)	56 (84.9)	0.565
Genital examination	59 (86.8)	55 (83.3)	0.436
Collection of blood samples	58 (85.3)	58 (87.9)	0.661
Repeated HIV testing	68 (100.0)	63 (95.5)	0.075
Collection of genital samples	59 (86.8)	58 (87.9)	0.846
Reimbursement	64 (94.1)	65 (98.5)	0.182
Other study activities^a^	53 (91.4)	52 (91.2)	0.977
Clinical services			
Visit schedules	64 (86.4)	57 (94.1)	0.129
Travel time to clinic	54 (79.4)	55 (83.3)	0.560
Clean clinic environment	68 (100.0)	65 (98.5)	0.308
Waiting time at clinic	67 (98.5)	62 (93.9)	0.161
Clinic staff attitude	68 (100.0)	65 (98.5)	0.308
Examination by female nurse	48 (70.6)	51 (77.3)	0.379
Examination by male nurse	68 (100.0)	63 (95.5)	0.075
Free treatment and condoms	67 (98.5)	64 (97.0)	0.542
Free counselling, health information	68 (100.0)	64 (98.5)	0.305
Hepatitis B vaccination^b^	61 (91.0)	-	-

^a^Other activities included in-depth interviews, focus group discussions and home visits’; ^b^only for those who received the surrogate vaccine; †chi-square test used to calculate *P* value

In terms of the clinical services and visits, in both groups, being examined by a male nurse was viewed as more acceptable than a female one. All men in the IV group liked being examined by a male nurse, while these views were not universal among men in the DV group (96%; *P* = 0.075). Other highly preferred aspects of the clinical services in both groups (>90% participants scored item as 4 or 5) were: clean clinic environment, clinic staff attitudes, free treatment and condoms, and counselling and health information, and the hepatitis B vaccine itself. Only 79% of the IV and 83% of the DV group scored travel time favourably (*P* = 0.560).

No associations were detected between the acceptability items and having attended all four study visits (Additional file 2: Table S2). Attendance levels among those who held less favourable views on the clinical services were high. Though differences were not significant, on 8 of the 10 measures of clinical services, those with less favourable views had a higher attendance than those with more favourable perceptions.

WTP in a future HIV vaccine trial was high in both groups (64/68, 94% in IV vs. 62/63, 98% in DV; *P* = 0.200). The main motivations for participation were potential HIV protection (81%), to help find a vaccine that works (75%), and to help others (68%). Interestingly, only 2% reported that reimbursements for study visits would motivate participation. Free HIV testing and treatment, and knowing someone with HIV were also not regarded as incentives for participation. No variations by study group were observed in these views. Almost all viewed side effects as a major concern for future trial participation (98%).

When asked about future use of an effective HIV vaccine, essentially all reported that they would accept this vaccine for themselves or their children. The most important attribute favouring vaccine acceptance was durability of protection (93/131, 70%). Few viewed ease of access (20/131, 15%), side effects (12/131, 9%), cost (3/131, 2%), number of doses (0/131, 0%) and duration that the vaccine was on the market (3/131, 2%) as important factors influencing whether they would accept an HIV vaccine known to be effective. Again, findings were similar between the two study arms.

## Discussion

This study indicates that it is feasible to recruit and retain a population of high-risk heterosexual men in an HIV vaccine trial in Johannesburg, South Africa. Recruitment was relatively efficient, with a screen-to-enrol ratio under two. Compared to non-eligible men, however, those enrolled in the study had lower risk behaviours and consequently are likely to have fewer incident HIV infections. This phenomenon raises the sample size required for demonstrating efficacy of an intervention [29]. Follow-up rates, approximately equal in both arms, were higher than in many previous vaccine preparedness studies among men [22].

In both study arms, the levels of WTP in an HIV trial were among the highest recorded among men in similar studies to date. In a review of 16 preparedness studies [22], willingness ranged from 40–99.4%, and was lower among men than women in most [22, 24, 30], but not all studies [31]. More generally, men are often less engaged in health care than women – which is commonly attributed to gendered social behaviours, occupational obligations and even a disinterest in their own health [32, 33] – and this may influence their decision to participate in trials. It was thus noteworthy to observe that the study population were highly motivated to enrol in future trials. Similar to other studies, altruistic motives often underlined this WTP, expressed as a desire to help find an efficacious vaccine and thereby contribute to improving the health of others and the greater community [10, 20, 21, 24, 25, 34, 35]. Though men looked very favourably upon reimbursements, they said that these would not motivate them to join a trial. This contrasts with commonly held views and some evidence in other studies in SSA that monetary incentives raise participation [36, 37].

Use of a surrogate vaccine and randomisation into immediate and deferred vaccine groups provided useful insights. Most especially, we were able to evaluate the relative acceptability of the different components of a future vaccine trial. The levels of discontent about the informed consent procedures in the IV arm suggest that participants require more information during consent relating to the vaccine and study procedures. Consent also needs to focus on ensuring participants comprehend the concepts of randomisation and unknown efficacy to avoid therapeutic misconception and potential increases in risk behaviours.

Also, in this study, as in others [21, 25], participants raised concerns about randomisation, particularly to a control or placebo group. Further, it is evident that many attributes of a study clinic, such as cleanliness and clinic staff attitudes, were highly valued by participants and likely are key determinants of retention in future trials. Notably, men placed a high premium on examination by a male health worker. The study design allowed us to demonstrate low return rates in the DV group for vaccination at month 12 when the vaccine was to be given to this group. This may have been because financial reimbursements and other clinical services were not provided at these visits. The latter two factors are perhaps more important than access to a technology, such as a vaccine, per se. While seemingly not a major barrier to participation, collection of specimens and travel distance to study clinics were important considerations for participants.

Even though men in the IV group experienced higher rates of adverse events than those allocated to DV, cohort retention was similar between study arms. While these events were all minor, this finding seemingly contrasts with that of a systematic review of qualitative studies on participation in HIV vaccine trials [38]. That review noted that vaccine safety was a foremost concern among potential trial participants (as in our study), but that this might undermine retention. Quite plausibly, however, patients who are concerned about adverse events might actually attend study visits to seek reassurance about their health, and therefore have high retention, as in this study.

A potential concern for all randomised blinded placebo controlled trials of HIV preventive technologies is the possibility that participants believe themselves to be protected against HIV, despite the vaccine’s unknown efficacy and the possibility that they were randomised to the placebo arm [16, 18]. In fact, many of the men in this study cited the potential for a vaccine to protect themselves from HIV infection as the principal motivator for joining a trial. In an HIV vaccine trial in Thailand, young recruits reduced condom use as their perception of HIV risk was lowered due to the vaccine [20]. By contrast, as in many other HIV prevention studies [3, 17, 19, 20, 35], risk activity actually decreased early on in our study in both groups. Overall, given the reduction in risk behaviours over time and fewer number of HIV infections in the IV group, this study does not support concerns about adverse changes in risk behaviour within HIV vaccine trials [39]. Nonetheless, counselling for participants in HIV trials in this setting should strongly address misconceptions about the efficacy of the candidate product [19, 20].

Overall, we contend that the use of randomisation, an actual vaccine and an untreated control group extends to knowledge beyond previous hypothetical vaccine studies. However, though the study findings might help optimise the design of future HIV vaccine trials, recruitment and retention rates in this study may not necessarily reflect those of a future trial. Firstly, the hepatitis B vaccine is of proven efficacy and hepatitis B differs from HIV in many important ways. Moreover, self report of WTP does not necessarily translate into participation in an HIV vaccine trial [10, 22, 24]. Other study limitations also bear mention. The small sample size constrained our ability to explore differences between groups, predictors of WTP and acceptability. Further, the majority of the risk behaviour responses were self-reported and influenced by social desirability and other biases. Participants, however, rated their responses as mostly truthful and we were able to triangulate their behavioural responses with HIV incidence, an objective marker of ongoing risk.

## Conclusions

Despite its limitations, the study was able to demonstrate a high degree of WTP in future trials, as shown by the actual retention rates and views expressed on the issue. Recruitment of men into vaccine trials that address the concerns raised here is therefore likely to be feasible and acceptable in this setting. While access to vaccines was an important motivation for enrolment in a future trial, quality health services were viewed as equally important, especially those provided by a male health worker. Moreover, the differences noted between study groups in sexual behaviour shows that changes in sexual behaviours might be anticipated in a vaccine trial in this population. Lastly, use of a surrogate vaccine, unlike previous studies based on hypothetical acceptability [22, 24, 40], replicates actual vaccine trial procedures and may enhance the ability of participants to accurately assess the perceived benefits and realistic consequences of participation.

## Additional files

Additional file 1: Table S1. Factors associated with participant retention; a generalised estimating equations analysis. (DOCX 28 kb)

Additional file 2: Table S2. Association between perspectives on acceptability collected at 12 months and attendance at all four study visits. (DOCX 29 kb)

## Abbreviations

AE: Adverse events
ART: Antiretroviral treatment
DV: Deferred vaccination
EDTCP: European and Developing Countries Clinical Trials Partnership
HIV: Human immuno-deficiency virus
IV: Immediate vaccination
SSA: Sub-Saharan Africa
STI: Sexually transmitted Infection
WTP: Willingness to participate
