# Supplementary material for: Using surrogate vaccines to assess feasibility and acceptability of future HIV vaccine trials in men: a randomised trial in inner-city Johannesburg, South Africa
Source: BMC Public Health. 2017 Jul 4;17(Suppl 3):113–22. doi: 10.1186/s12889-017-4355-z (PMC5498868; doi:10.1186/s12889-017-4355-z)
Supplement: Supplementary file 1 — Factors associated with participant retention; a generalised estimating equations analysis. (DOCX 28 kb) [file 12889_2017_4355_MOESM1_ESM.docx]

**Additional file 1: Table S1: Factors associated with participant retention; a generalised estimating equations analysis**

| **Variable** | **Unadjusted OR** | ***P*** | **Adjusted OR** | ***P*** |
| --- | --- | --- | --- | --- |
| Trial group  Deferred vaccination  Immediate vaccination | 1.0  1.30 (0.51-3.32) | 0.583 | 1.0  1.36 (0.56-3.35) | 0.502 |
| Age (years)  18-24  25-34  >=35 | 1.0  1.68 (0.58-4.85)  1.38 (0.40-4.80) | 0.335  0.608 | 1.0  1.58 (0.55-4.61)  1.33 (0.37-4.86) | 0.394  0.659 |
| Born in SA  Yes  No | 1.0  1.30 (0.46-3.70) | 0.626 | - | - |
| Single  Yes  No | 1.0  1.78 (0.48-6.60) | 0.389 | - | - |
| Any employment^‡‡^  Yes  No | 1.0  0.62 (0.24-1.60) | 0.327 | - | - |
| Completed secondary education  Yes  No | 1.0  0.67 (0.26-1.71) | 0.397 | - | - |
| Johannesburg resident >1 year  Yes  No | 1.0  0.55 (0.13-2.24) | 0.400 | - | - |
| >1 partner in past 3 months  Yes  No | 1.0  0.67 (0.22-2.03) | 0.483 | - | - |
| Consistent condom use  Yes  No | 1.0  0.61 (0.20-1.83) | 0.376 | - | - |
| High perceived HIV risk  Yes  No | 1.0  1.22 (0.41-.3.61) | 0.722 | - | - |
| Previous sex with male partner  Yes  No | 1.0  3.78 (0.88-16.16) | 0.073 | 1.0  3.12 (0.59-16.51) | 0.180 |
| Circumcised  Yes  No | 1.0  1.68 (0.65-4.38) | 0.287 | - | - |
| Laboratory STI at baseline  Yes  No | 1.0  1.19 (0.33-4.30) | 0.792 | - | - |
| Adverse event  No  Yes | 1.0  1.35 (1.13-1.62) | 0.001 | 1.0  3.76 (0.82-17.14) | 0.087 |

OR Odds ratio, ^‡‡^Part-time, self- or full-employment
