# Supplementary material for: Using surrogate vaccines to assess feasibility and acceptability of future HIV vaccine trials in men: a randomised trial in inner-city Johannesburg, South Africa
Source: BMC Public Health. 2017 Jul 4;17(Suppl 3):113–22. doi: 10.1186/s12889-017-4355-z (PMC5498868; doi:10.1186/s12889-017-4355-z)
Supplement: Supplementary file 2 — Association between perspectives on acceptability collected at 12 months and attendance at all four study visits. (DOCX 29 kb) [file 12889_2017_4355_MOESM2_ESM.docx]

**Additional file 2: Table S2: Association between perspectives on acceptability collected at 12 months and attendance at all four study visits**

| **Variable** | **Proportion attending all visits (n=124)** | ***P*** |
| --- | --- | --- |
| **Study procedures** | | |
| Informed consent  Scored 0-3  Scored 4-5 | 17/17 (100.0%)  107/117 (91.5%) | 0.361 |
| Randomisation  Scored 0-3  Scored 4-5 | 28/30 (93.3%)  96/104 (92.3%) | 0.605 |
| Completing questionnaires  Scored 0-3  Scored 4-5 | 9/10 (90.0%)  115/123 (93.5%) | 0.516 |
| Physical examination  Scored 0-3  Scored 4-5 | 15/18 (88.3%)  109/116 (94.0%) | 0.133 |
| Genital examination  Scored 0-3  Scored 4-5 | 17/20 (85%)  107/114 (93.9%) | 0.171 |
| Collection of blood samples  Scored 0-3  Scored 4-5 | 18/18 (100.0%)  106/116 (91.4%) | 0.224 |
| Repeated HIV testing  Scored 0-3  Scored 4-5 | 3/3 (100.0%)  121/131 (92.4%) | 0.791 |
| Collection of genital samples  Scored 0-3  Scored 4-5 | 16/17 (94.1%)  108/107 (92.3%) | 0.630 |
| Reimbursement  Scored 0-3  Scored 4-5 | 5/5 (100.0%)  118/128 (92.2%) | 0.672 |
| Other study activities*  Scored 0-3  Scored 4-5 | 8/10 (80.0%)  99/105 (94.3%) | 0.144 |
| Clinical services | | |
| Visit schedules  Scored 0-3  Scored 4-5 | 12/13 (92.3%)  112/121 (92.6%) | 0.653 |
| Travel time to clinic  Scored 0-3  Scored 4-5 | 22/25 (88.0%)  102/109 (93.6%) | 0.395 |
| Clean clinic environment  Scored 0-3  Scored 4-5 | 1/1 (100.0%)  123/133 (92.5%) | 0.925 |
| Waiting time at clinic  Scored 0-3  Scored 4-5 | 4/5 (100.0%)  120/129 (93.0%) | 0.326 |
| Clinic staff attitude  Scored 0-3  Scored 4-5 | 1/1 (100.0%)  123/133 (92.5%) | 0.925 |
| Exam by female nurse  Scored 0-3  Scored 4-5 | 33/35 (94.3%)  91/99 (91.9%) | 0.488 |
| Exam by male nurse  Scored 0-3  Scored 4-5 | 3/3 (100.0%)  121/131 (92.4%) | 0.791 |
| Free treatment, condoms  Scored 0-3  Scored 4-5 | 3/3 (100.0%)  121/131 (92.4%) | 0.791 |
| Free counselling, health info  Scored 0-3  Scored 4-5 | 1/1 (100.0%)  122/132 (92.5%) | 0.925 |
| Hepatitis B vaccination**  Scored 0-3  Scored 4-5 | 6/6 (100.0%)  54/61 (88.5%) | 0.502 |

Table shows the proportion who attended all four study visits, by each acceptability item. *Other activities included in-depth interviews, focus group discussions and home visits’; **only for those who received the surrogate vaccine; chi-square test used to calculate *P* values
